# Trends in substance use and associations with internet use among Japanese high school students: A nationwide survey, 2018–2024

**DOI:** 10.1002/pcn5.70365

**Published:** 2026-06-17

**Authors:** Satomi Mizuno, Satoshi Inoura, Maki Kitamura, Toshihiko Matsumoto, Kunihiko Kitagaki, Akihiro Koide, Kenji Takehara, Takuya Shimane

**Affiliations:** ^1^ Department of Drug Dependence Research, National Institute of Mental Health National Center of Neurology and Psychiatry Kodaira Tokyo Japan; ^2^ Department of Nursing, Faculty of Nursing Niigata Seiryo University Niigata Japan; ^3^ Social Pharmacy Laboratory, School of Pharmacy Tokyo University of Pharmacy and Life Sciences Hachioji Japan; ^4^ Regulatory Science Laboratory Yokohama University of Pharmacy Yokohama Japan; ^5^ Department of Health Policy National Centre for Child Health and Development Tokyo Japan

**Keywords:** adolescents, high school students, internet use, nationwide survey, substance use

## Abstract

**Aim:**

To examine temporal trends in past‐year substance use among Japanese high school students and their associations with daily time spent on internet activities.

**Methods:**

We analyzed pooled data from three nationwide repeated cross‐sectional surveys conducted in 2018, 2021, and 2024, including 142,567 Japanese high school students aged 16–18 years. Past‐year substance use (illicit drug and alcohol use and tobacco smoking) and daily time spent on internet activities (social networking services, online gaming, internet searching, video or music streaming, and online shopping or auctions) were assessed using self‐administered questionnaires. Associations between internet use and substance use were examined using survey‐weighted logistic regression models with restricted cubic spline functions to assess nonlinear patterns across exposure levels.

**Results:**

Past‐year illicit drug use remained rare across the survey years (0.1%–0.3%). Alcohol use declined from 14.7% in 2018 to 8.0% in 2024, whereas tobacco smoking decreased from 1.8% to 1.3%. For illicit drug use, probabilities remained low at lower exposure levels but increased at higher levels, most notably for online shopping or auctions. Regarding alcohol use, a longer time spent on all internet activities was associated with higher probabilities, with steeper increases observed for social networking services and online shopping or auctions at higher exposure levels. For tobacco smoking, positive associations were most evident with networking services, online shopping, and auctions.

**Conclusion:**

Activity‐specific patterns indicate heterogeneity in the associations between internet and substance use, highlighting the importance of considering the types of online activities alongside overall internet use.

## INTRODUCTION

Substance use by adolescents has long been recognized as an important public health concern, owing to its potential implications for physical health, mental well‐being, and social development.^1^, ^2^, ^3^, ^4^, ^5^ In many Western countries, illicit drug and alcohol use and tobacco smoking are relatively common among adolescents, and long‐term surveillance has documented substantial temporal changes in their prevalence.^6^, ^7^, ^8^ By contrast, substance use among Japanese high school students has consistently been reported at much lower levels, particularly with respect to illicit drugs.^9^, ^10^, ^11^, ^12^

Although this low‐prevalence context is often regarded as favorable, nationally representative evidence on recent temporal trends in adolescent substance use in Japan remains limited. Previous studies have provided valuable insights into trends in alcohol use and tobacco smoking using repeated surveys.^9^ However, illicit drug use has been frequently excluded from trend analyses because of its rarity and inconsistent measurements across survey waves.^11^ Consequently, little is known about how illicit drug use, alcohol use, and tobacco smoking have evolved concurrently within a unified analytic framework using the same nationally representative samples.

During the same period, adolescents' daily environments have changed substantially with the widespread adoption of the internet. Internet use among adolescents has diversified beyond overall screen time to encompass a range of activities, including social networking services (SNS), online gaming, information searching, video or music streaming, and online shopping.^3^, ^13^, ^14^, ^15^ These activity‐specific patterns may reflect distinct social contexts and daily routines that may be differentially related to substance‐use behavior.^16^, ^17^ Nevertheless, most existing studies have focused on overall internet use or single activities, and few have examined multiple substance use outcomes in relation to activity‐specific internet use,^18^, ^19^, ^20^, ^21^, ^22^, ^23^ particularly in low‐prevalence settings such as Japan.^10^

To build on prior work and address these gaps, we analyzed pooled data from three nationwide repeated cross‐sectional surveys conducted in 2018, 2021, and 2024 to describe recent trends in past‐year illicit drug use, alcohol use, and tobacco smoking among Japanese high school students using harmonized measures and to examine whether time spent on specific internet activities shows similar or distinct associations across substance use outcomes.

## METHODS

### Survey design and target population

Data from three repeated cross‐sectional school‐based surveys conducted in 2018, 2021, and 2024 were used. Each wave employed a self‐administered, anonymous questionnaire distributed to high school students (typically aged 16–18 years) from a nationally representative school sample.

### Stratified cluster sampling methodology

All students enrolled in randomly selected high schools in Japan were eligible to participate. According to the national school database used for sampling, approximately 121,492–193,543 students were enrolled in 4674–4736 public, private, and national high schools in each survey year. A stratified one‐stage cluster sampling design was employed in which all students within the selected schools were invited to participate. Stratification and sampling frames varied by survey year. In the 2018 and 2021 surveys, sampling and stratification were conducted using six geographic blocks (Hokkaido‐Tohoku, Kanto, Hokuriku‐Tokai, Kinki, Chugoku‐Shikoku, and Kyushu‐Okinawa). In the 2024 survey, the sampling frame was defined at the prefecture level (all 47 prefectures), whereas stratification was based on the same six geographic blocks used in the earlier surveys. Within each stratum, schools were selected using probability proportional to size sampling based on student enrollment, allowing schools of different sizes to be appropriately represented. During the survey period, 82–236 high schools were randomly selected from the national school database. Sampling weights were calculated separately for each survey year to reflect the differences in sampling probabilities arising from year‐specific stratification schemes.

### Survey implementation procedures

The survey procedures were conducted using the same standardized protocol as in our previous nationwide survey.^11^, ^24^ Surveys were conducted between September and March for each wave. Explanatory materials were distributed to the selected schools, and parents or guardians were informed through school‐mediated discoveries.

Data were collected using self‐administered questionnaires that were completed during regular school hours. Paper‐based questionnaires were used in 2018 and 2021, and a web‐based option was introduced in 2024, at the request of some schools. Participation was voluntary and anonymous. Informed consent was obtained at the beginning of the questionnaire.

The data entry and initial processing were outsourced under strict confidentiality agreements. School names were not collected, and analyses were conducted using anonymized school identifiers only.

### Ethical considerations

All survey protocols were approved by the Ethics Committee (2018/2021: A2018‐055; 2024: A2023‐123), and informed consent was obtained from all students using procedures consistent with those of previous studies,^11^, ^24^ in accordance with the Declaration of Helsinki.

### Study population

The analytical sample was defined using prespecified exclusion criteria. Students were excluded if the information required for the complex sampling design (e.g., survey year, sampling weight, stratum, or school identifier) was missing; if questionnaires were blank or had less than 50% of the items completed; if students were enrolled in special education programs; or if data on sex, grade, geographic area, or survey mode required for standardization were missing. Participants reporting “other gender” were excluded from the primary analyses to ensure comparability across survey years, given substantial differences in sample size across waves. Although this approach improves comparability, it may limit the generalizability of the findings to sexually diverse adolescents. Missing data were not imputed because their proportion was small (<3% for the variables used in the main analyses).

### Survey items and variables

Each survey wave included 43–45 questionnaire items, with minor variations across the years. In this study, analyses were restricted to items that were identically worded and consistently available across all survey waves conducted in 2018, 2021, and 2024 to ensure comparability across survey years. The original Japanese wording for all questionnaire items was published in a previous national survey report.^24^

The primary outcome variable was self‐reported past‐year substance use, assessed separately for illicit drug and alcohol use and tobacco smoking. Past‐year illicit drug use was defined as any self‐reported use of cannabis, inhalants, methamphetamine, new psychoactive substances, cocaine, or 3,4‐methylenedioxymethamphetamine (MDMA) in the preceding 12 months. Past‐year alcohol use and past‐year tobacco smoking were defined analogously as any use within the same timeframe. All substance use outcomes were analyzed as binary variables (yes/no).

The main independent variables were patterns of internet use. Respondents reported their average daily time spent using the internet during the past 30 days on five distinct activities: SNS, online gaming, internet searching or information browsing, watching videos or listening to music online, and online shopping, including auctions. For each activity, respondents selected one of six ordered response categories: no use (0 min), less than 30 min, approximately 1 h, approximately 1–3 h, approximately 4–5 h, and ≥6 h. These variables were analyzed both as ordinal measures, coded from 0 to 5 to assess monotonic dose–response trends, and as categorical variables with “no use” as the reference category in category‐specific and sensitivity analyses. Category‐specific models were used to explore potential nonlinearity.

To address potential confounding factors, covariates were selected a priori based on their theoretical relevance and previous epidemiological studies.^11^ The demographic characteristics included sex, grade, geographic area, and survey year, all of which were treated as core adjustment variables in every model. The survey mode (paper‐based or online) was also included as a covariate to account for potential differences in reporting associated with the data collection procedures.

Substance‐related attitudes were included as covariates. These comprised permissive attitudes toward drug use. In the analyses focusing on alcohol and tobacco outcomes, permissive attitudes toward the corresponding substances were included to ensure conceptual consistency between the outcome and adjustment variables.

Time without adult supervision was included as an indicator of reduced supervision and opportunities for risky behavior. Based on prior Japanese research,^25^ this variable was operationalized as a dichotomous indicator representing long unsupervised time (≥3 h per day vs. <3 h).

Social relationships were assessed using items capturing everyday peer interactions and emotional support, including dissatisfaction with school life, lack of close friends with whom to interact, and infrequent consultations with parents.^16^, ^25^ These measures were included to account for social connectedness and support, which have been shown to be associated with adolescent risk behaviors in previous studies.

### Data cleaning procedures

Substance use variables were cleaned using the same predefined consistency rules as in our previous nationwide surveys,^11^, ^24^, ^25^ which were applied identically across survey years. The corrections were minimal (approximately 0.03% per wave).

### Statistical analyses

All analyses accounted for a complex survey design incorporating sampling weights, stratification, and clustering at the school level. Data from three nationally representative surveys conducted in 2018, 2021, and 2024 were pooled, with schools treated as the primary sampling units. To ensure appropriate variance estimation when pooling the survey waves, the strata were defined as the cross‐classification of the survey year and geographical area. The sampling weights were constructed separately for each survey year to represent the national population of Japanese high school students.

Survey‐weighted prevalence estimates and 95% confidence intervals (CIs) were calculated for past‐year illicit drug, alcohol, and tobacco use. To facilitate comparisons across survey years, prevalence estimates were directly standardized to the covariate distribution of the 2024 survey population using marginal standardization based on survey‐weighted logistic regression models adjusted for grade, sex, geographic area, and survey mode. Individual‐level predicted probabilities were averaged over the 2024 standard population, incorporating sampling weights, and variance estimation accounted for stratification and clustering using design‐based methods.

Differences in substance use prevalence across survey years were evaluated using survey‐weighted logistic regression models, with the survey year entered as a categorical variable, and the overall year effect was assessed using a Wald‐type test. Year‐to‐year differences in the distribution of time spent on internet activities were evaluated using Rao–Scott design‐adjusted tests. Linear trends were assessed by modeling the survey year as an ordinal variable, and year‐specific differences relative to the most recent survey (2024) were estimated using survey‐weighted logistic regression models, with the survey year entered as a categorical variable.

Associations between the internet and substance use were examined using survey‐weighted generalized linear models with a quasibinomial link. Internet use reflected typical daily activity at the time of the survey, whereas substance use outcomes referred to any use within the past 12 months. Therefore, all analyses were explicitly framed as describing cross‐sectional associations rather than temporal or causal relationships.

For the primary analyses, the time spent on each type of internet activity was treated as ordinal exposure with six ordered categories (coded 0–5) and modeled using restricted cubic spline functions with linear tails. In practice, the spline terms are implemented using natural cubic spline basis functions (ns) within survey‐weighted logistic regression models (svyglm). Boundary knots were fixed at the minimum and maximum exposure categories (0 and 5), and interior knots were placed at the survey‐weighted percentiles (10th, 50th, and 90th) of the exposure distribution for each activity to allow flexible modeling of nonlinear patterns across ordered categories. The lowest exposure category (no use) was used as the reference.

To summarize the associations on an absolute scale, marginal predicted probabilities of substance use were estimated for each exposure category by fixing the exposure level and averaging the predicted probabilities over observed covariates standardized to the 2024 survey population. For the spline curves, point estimates were obtained from primary survey‐weighted models. The uncertainty of category‐specific predicted probabilities was quantified using bootstrap replicate weights (400 replicates), with models refitted within each replicate and 95% CI calculated from replicate‐based standard errors. Replicate‐weight methods were used for the uncertainty estimation of the model‐based predicted probabilities, as shown in Figure 2, whereas CIs for regression coefficients and odds ratios were obtained using standard design‐based variance estimation from svyglm.

As a secondary analysis, internet use time was additionally modeled as an ordinal variable to estimate adjusted odds ratios representing the average change in odds associated with a one‐category increase in use time. Category‐specific models, using no use as the reference group, were also fitted to describe the associations at specific exposure levels. Given the large number of statistical tests arising from multiple internet activities, substance use outcomes, and modeling approaches, the analyses were structured hierarchically. Spline‐based models examining activity‐specific patterns were prespecified as the primary analyses, with emphasis placed on the overall shape and concentration of associations rather than individual *p*‐values. Secondary analyses were descriptive and exploratory, and *p*‐values were interpreted cautiously in conjunction with effect size patterns and consistency across analyses.

Sensitivity analyses were additionally adjusted for psychosocial and behavioral factors, recognizing that these variables may reflect downstream or intermediate processes rather than core confounders. Category‐specific models were fitted as sensitivity analyses to provide easily interpretable contrasts relative to non‐use and to corroborate the patterns observed in spline‐based models. Sensitivity‐ and category‐specific analyses were conducted to assess the robustness and interpretability of the primary spline‐based findings and were not intended for formal hypothesis testing. Exploratory sex‐stratified analyses were also conducted using ordinal trend models to assess the consistency of associations between internet activities and substance use across sexes.

All analyses were conducted using complete‐case data in R software version 4.4.3 (R Foundation for Statistical Computing, Vienna, Austria) with the survey package. *p*‐values < 0.05 were considered significant.

## RESULTS

**Figure 1:**
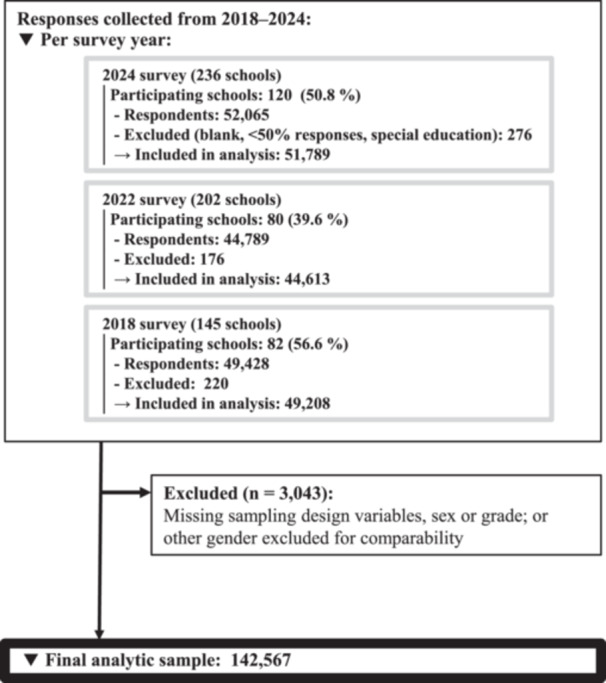
Flowchart of participant selection in the nationwide high school survey (2018–2024).

Between 2018 and 2024, 145,610 students participated in three repeated nationwide cross‐sectional surveys. After excluding 3043 students with insufficient responses, missing, or non‐binary sex data, 142,567 students were included in the final analysis (Figure 1). Participant characteristics according to the survey year are shown in Table S1. The sampling weights reflected the inverse probabilities of school selection and student responses (Table S2). The distribution of final weights was right‐skewed (median 1485; interquartile range 583–9832), and design effects were substantially larger for internet use variables than for substance use outcomes, indicating notable clustering and weighting effects for behavioral exposures (Tables S3 and S4). The effective sample sizes for internet use variables were substantially reduced owing to clustering, as reflected by the large design effects.

### Trends in substance use

The survey‐weighted crude and standardized prevalence estimates are presented in Tables 1 and S5. Standardized prevalence estimates are emphasized in the main text. In the past year, illicit drug use remained rare throughout the study period (standardized prevalence: 0.3%, 0.1%, and 0.2% in 2018, 2021, and 2024, respectively). Although the overall effect of survey year was significant (Wald *p* = 0.007), the year‐specific differences relative to 2024 were small and imprecise (Table 2). In contrast, alcohol use in the past year has declined steadily over time, with the standardized prevalence decreasing from 14.7% in 2018 to 8.0% in 2024. Past‐year tobacco smoking also declined between 2018 and 2021 and remained low thereafter, although the adjusted pairwise comparisons relative to 2024 did not reach significance (Table 2).

**Table 1:** Survey‐weighted prevalence standardized to the 2024 covariate distribution (%) of substance use by survey year.

	2018 % (95% CI)	2021 % (95% CI)	2024 % (95% CI)
Past‐year substance use			
Illicit drugs	0.3 (0.2–0.5)	0.1 (0.1–0.2)	0.2 (0.1–0.2)
Alcohol	14.7 (12.8–16.8)	9.8 (8.4–11.4)	8.0 (7.3–8.7)
Tobacco	1.8 (1.3–2.6)	0.9 (0.7–1.3)	1.3 (1.1–1.5)

*Note*: Estimates are survey‐weighted and standardized to the covariate distribution of the 2024 survey population (grade, sex, area, and survey mode [paper]). 95% CIs were calculated using design‐based variance estimation accounting for stratification and clustering, as implemented in survey‐weighted regression models. The standardized prevalence was obtained from survey‐weighted logistic regression models, including the survey year and covariates, and averaged over the 2024 standard population. The survey mode differed by year; 2018 and 2021 were paper‐based, whereas 2024 included both paper and web responses.

Abbreviation: 95% CI, 95% confidence interval.

**Table 2:** Adjusted odds ratios for substance use outcomes by survey year, adjusted for demographic characteristics and survey design (reference: 2024).

Outcome	Overall year effect (Wald *p*‐value)	2018 versus 2024 aOR (95% CI), *p*	2021 versus 2024 aOR (95% CI), *p*
Past‐year substance use			
Illicit drugs	*p* = 0.007	1.63 (0.99–2.68), *p* = 0.057	0.75 (0.43–1.33), *p* = 0.332
Alcohol	*p* < 0.001	1.98 (1.63–2.41), *p* < 0.001	1.25 (1.02–1.53), *p* = 0.036
Tobacco	*p* = 0.003	1.41 (0.94–2.10), *p* = 0.096	0.72 (0.48–1.09), *p* = 0.122

*Note*: aORs and 95% CIs were estimated using survey‐weighted logistic regression models (svyglm with quasibinomial family), accounting for clustering by school (PSU), stratification by area, and sampling weights (survey.lonely.psu = “adjust”). The models were adjusted for grade, sex, area, and survey mode (paper/online). The 2024 survey year was used as the reference category. The overall effect of the survey year (three‐level categorical variables: 2018, 2021, and 2024) was assessed using the Wald‐type test.

Abbreviations: aOR, adjusted odds ratio; CI, confidence interval.

### Trends in internet use

The distribution of time spent on internet activities by survey year is summarized in Tables 3 and S5. Although the overall distributions differed across survey years (Table 3A), the linear trends were modest and did not reach statistical significance for SNS and online gaming, indicating heterogeneous rather than monotonic changes across time categories (Table 3B). The time spent watching videos or listening to music online, and the time spent on online shopping or auctions, increased over time. In contrast, the time spent searching the internet or browsing information showed a modest decline over time.

**Table 3:** Changes in time spent on internet activities across survey years.

A. Year‐to‐year differences in the distribution of time spent on internet activities (Rao–Scott design‐adjusted *F* test)				
Internet activity (6‐level)	Rao–Scott *F* statistic	df (numerator)	df (denominator)	*p*‐value
Time spent on SNS	6.71	3.46	940.5	<0.001
Time spent on online gaming	2.48	3.02	821.2	0.059
Time spent on internet searching/information browsing	6.99	4.77	1296.6	<0.001
Time spent watching videos or listening to music online	11.04	3.07	833.9	<0.001
Time spent on online shopping or auctions	8.11	2.94	801.0	<0.001

B. Adjusted linear trends in time spent on internet activities across survey years (survey‐weighted ordinal logistic regression)		
Internet activity (6‐level ordered)	Trend aOR per year step (95% CI)	*p*‐value
Time spent on SNS	1.11 (0.99–1.23)	0.060
Time spent on online gaming	1.08 (0.97–1.20)	0.157
Time spent on internet searching/information browsing	0.94 (0.89–0.99)	0.018
Time spent watching videos or listening to music online	1.34 (1.20–1.50)	<0.001
Time spent on online shopping or auctions	1.26 (1.13–1.39)	<0.001

*Note*: Table 3A shows whether the distribution of time spent on each internet activity differed across the survey years (2018, 2021, and 2024). Test: Rao–Scott design‐adjusted chi‐square test expressed as an *F* statistic (svychisq, statistic = “F”). This test evaluates whether the overall distribution of a 6‐level ordinal variable differs across the survey years. The test does not indicate the direction of change (increase or decrease). Table 3B shows the direction and magnitude of the linear trends in the time spent on internet activities over time. Model: survey‐weighted ordinal logistic regression (proportional odds model). The survey year was coded as a linear trend variable (2018 = 0, 2021 = 1, and 2024 = 2). The aOR represents the change in the odds of being in a higher time category per survey step (e.g., 2018 → 2021). aOR > 1 indicates a shift toward longer time spent, and aOR < 1 indicates a shift toward shorter time spent. Adjusted for grade, sex, area, and survey mode (papers/online). Survey design accounted for clustering by school (PSU), stratification by area, and sampling weights (survey.lonely.psu = “adjust”).

Abbreviations: aOR, adjusted odds ratio; CI, confidence interval; SNS, social networking services.

### Associations between the internet and substance use

**Figure 2:**
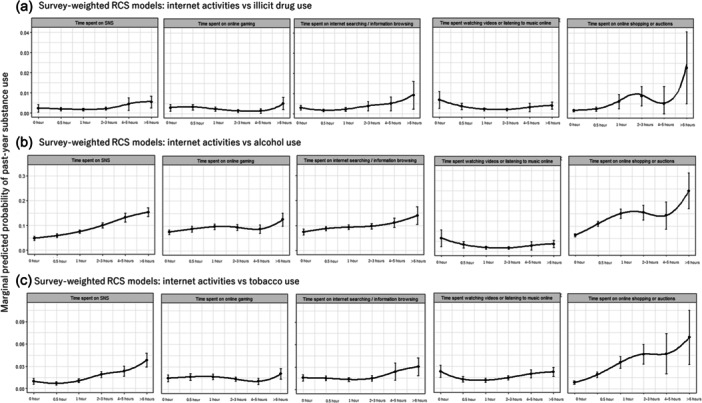
Survey‐weighted spline models of internet use time and past‐year substance use among Japanese high school students. Survey‐weighted spline models showing marginal predicted probabilities of past‐year illicit drug (a) and alcohol use (b), and tobacco smoking (c) across ordinal categories of time spent on different internet activities (0–5). Solid lines represent point estimates from survey‐weighted spline models, and points indicate category‐specific marginal predicted probabilities. The vertical error bars denote design‐based 95% confidence intervals. All models were adjusted for survey year, grade, sex, geographic area, and survey mode, and accounted for clustering at the school level and stratification by area. The confidence intervals were wider for higher exposure categories because of the low prevalence of some outcomes.

Associations between daily internet use duration and past‐year substance use outcomes were primarily evaluated using restricted cubic spline models, which constituted the prespecified primary analytic approach for assessing nonlinear exposure–response relationships (Figure 2). Category‐specific odds ratios derived from the same spline models are provided in Tables S6–S8 to facilitate interpretation at discrete exposure levels. Ordinal trend models summarizing the average linear associations are presented in Table 4 to aid interpretation. Category‐specific estimates obtained from separate survey‐weighted logistic regression models using “no use” as the reference category are reported in Tables S9–S11. These secondary analyses were conducted to illustrate the estimated stability and data sparsity at extreme exposure levels and should be interpreted descriptively.

**Table 4:** Associations between internet use patterns and past‐year substance use among Japanese high school students.

Internet activity	Illicit drugs		Alcohol		Tobacco	
	aOR per 1‐category increase (95% CI)	*p*‐value	aOR per 1‐category increase (95% CI)	*p*‐value	aOR per 1‐category increase (95% CI)	*p*‐value
SNS	1.24 (1.05–1.48)	0.014	1.32 (1.27–1.36)	<0.001	1.46 (1.34–1.58)	<0.001
Online gaming	0.96 (0.81–1.14)	0.654	1.09 (1.04–1.14)	<0.001	1.00 (0.91–1.10)	0.995
Internet searching/information browsing	1.29 (1.08–1.54)	0.005	1.12 (1.07–1.17)	<0.001	1.08 (1.00–1.17)	0.044
Video/music	0.99 (0.82–1.19)	0.881	1.20 (1.15–1.26)	<0.001	1.16 (1.06–1.27)	0.002
Online shopping or auctions	1.72 (1.50–1.98)	<0.001	1.43 (1.37–1.50)	<0.001	1.66 (1.54–1.79)	<0.001

*Note*: Outcome variables were: past‐year illicit drug use, alcohol use, and tobacco use. Model: survey‐weighted logistic regression (svyglm, quasibinomial). Exposure variables: Time spent on internet activities was entered as an ordinal predictor (six ordered categories). Interpretation: Adjusted odds ratios (aORs) represent the change in the odds of substance use per category increase in the time spent on the activity. Covariates: Survey year (categorical; reference = 2024), grade, sex, area, and survey mode (paper/online). Survey design: clustered by school (PSU), stratified by area, with sampling weights applied (survey.lonely.psu = “adjust”).

Abbreviations: 95% CI, 95% confidence interval; aOR, adjusted odds ratio; SNS, social networking services.

Design‐based Wald tests comparing linear trends and spline specifications supported the overall associations for most activity–outcome pairs and provided evidence of nonlinearity for several relationships (Table S12). In some cases (e.g., online gaming and video/music for illicit drug use), overall spline associations were detected despite weak linear trend tests, underscoring the value of assessing nonlinear patterns.

For the past‐year illicit drug use, the associations varied by activity type. In the ordinal trend models (Table 4), the time spent on online shopping or auctions showed the strongest positive association, whereas smaller positive associations were observed for SNS, internet searching, and information browsing. In contrast, the time spent watching videos or listening to music online did not show a consistent positive dose–response pattern. Spline‐based analyses (Figure 2; Tables S6–S8) indicated that the predicted probabilities of illicit drug use remained low at lower exposure levels but tended to increase at higher levels of engagement, particularly for online shopping or auctions and prolonged internet searching. The estimates at extreme exposure levels were imprecise, with wider CIs reflecting sparse data in some categories, as illustrated by category‐specific models (Table S9) and the large design effects inherent to the complex survey design (Table S4). For watching videos or listening to music online, spline‐based estimates suggested lower odds at moderate use levels relative to non‐use, although uncertainty remained in the highest categories.

Longer time spent on internet activities was associated with higher odds of alcohol use in the past year (Table 4). Restricted cubic spline analyses showed largely monotonic increases in the predicted probabilities with longer daily engagement, with steeper increases for SNS and online shopping or auctions, particularly at ≥4 h per day (Figure 2).

For past‐year tobacco smoking, stronger associations were observed for SNS and online shopping or auctions, whereas the associations with other activities were weaker or inconsistent (Table 4). The restricted cubic spline analyses suggested a nonlinear pattern with elevated probabilities concentrated at higher exposure levels for these activities (Figure 2).

Because multiple associations were examined across activities, outcomes, and modeling approaches, some findings may reflect chance. Therefore, emphasis was placed on the consistency of patterns across analyses, rather than on individual *p*‐values. As a sensitivity check, a false discovery rate (Benjamini–Hochberg) adjustment was applied to the primary set of associations; the overall patterns and key conclusions remained unchanged (Table S13).

### Sensitivity analyses

After additional adjustments for substance‐related attitudes, school dissatisfaction, peer relationships, parental consultation, and unsupervised time, most of the associations with drug use were attenuated. However, the association with online shopping or auction usage persisted, particularly at higher exposure levels (Tables 4 and S14). For alcohol and tobacco use, associations with SNS and online shopping or auctions remained evident after additional adjustments, although the effect sizes were modestly reduced (Tables S14–S17).

### Exploratory sex‐stratified analyses

Exploratory sex‐stratified analyses showed overall patterns broadly similar to those derived in the pooled analyses presented in Table 4 (Table S18). In both males and females, engaging in online shopping or auctions showed consistently positive associations across all substance use outcomes. Associations with alcohol use were also generally similar across sexes for most internet activities. However, some activity‐specific differences were observed for illicit drug use, with associations for online gaming appearing more evident among females and associations for internet searching/information browsing appearing somewhat stronger among males.

## DISCUSSION

Using nationally representative data from three survey waves (2018–2024), we examined trends in adolescent substance use in Japan and their associations with the time spent on specific internet activities. Past illicit drug use remained rare across the survey years, whereas alcohol use and tobacco smoking declined. Importantly, associations with internet use differed by activity type, rather than following a uniform pattern across all online behaviors.

By focusing on activity‐specific internet use, this study extends prior research, which has largely relied on aggregated measures of overall use or single survey waves.^13^, ^14^, ^15^ To our knowledge, few nationally representative studies in low‐prevalence settings have simultaneously examined multiple substance use outcomes across repeated survey waves, while distinguishing between specific types of online activities. Most prior studies have either focused on total screen time or examined a single platform or behavior in isolation. By contrast, the present analysis integrates harmonized measures across three nationwide surveys and compares multiple internet activities within the same analytical framework. This approach allows for a direct assessment of whether distinct forms of online engagement demonstrate different patterns of association with substance use in a context where the overall prevalence remains low.

### Illicit drug use in a low‐prevalence context

Illicit drug use among Japanese high school students is consistently uncommon, with limited absolute year‐to‐year variation. Although statistical tests indicated a year effect, the magnitude of the change was small and likely reflected a large sample size rather than meaningful shifts in population prevalence. In low‐prevalence contexts, effect estimates are inherently imprecise, particularly at higher exposure levels.^11^ While some activity‐specific associations were observed in the primary models, they were attenuated in the sensitivity analyses and were accompanied by wide CIs. Notably, even at the highest exposure levels, the predicted absolute probability of drug use remained <1%, reflecting the overall rarity of the outcomes.

Therefore, these findings should not be interpreted as evidence that specific internet activities are directly linked to drug use. Rather, they likely reflect population‐level co‐occurrence within shared behavioral or environmental contexts.^3^, ^13^, ^18^, ^20^ The relatively consistent association observed between online shopping and auction usage should be interpreted cautiously. This may reflect unmeasured contextual or behavioral factors that co‐occur with both this type of online engagement and substance use, rather than a direct or content‐specific mechanism.

Despite the large nominal sample size, the effective sample size was reduced by clustering and weighting, resulting in substantial design effects. Consequently, the detailed features of the spline curves, particularly for rare outcomes, should be interpreted with caution. Nevertheless, the overall direction of the activity‐specific patterns was broadly consistent across survey years, modeling approaches, and sensitivity analyses.

### Alcohol use and tobacco smoking

Associations between internet use and alcohol use or tobacco smoking were observed more consistently. Longer time spent on activities involving social interaction or consumer‐oriented engagement, such as SNS and online shopping or auctions, was associated with a higher likelihood of alcohol or tobacco use after adjusting for demographic and psychosocial covariates.^10^, ^21^, ^26^, ^27^

Because both alcohol use and tobacco smoking are prohibited among high school students in Japan, these findings should not be interpreted as evidence that internet use promotes substance use.^1^, ^16^, ^17^ Instead, the observed associations likely reflect broader behavioral contexts in which multiple behaviors co‐occur. For example, adolescents who spend extended time in relatively unstructured settings may be more likely to engage in a range of risk‐related behaviors. Given the repeated cross‐sectional design, a causal interpretation is not warranted.

### Internet activities as contextual indicators of substance‐use risk

These findings on activity‐specific patterns and exposure–response relationships between internet use and substance use should be interpreted at two complementary levels. The association was most pronounced for online shopping or auctions, while more moderate and less consistent associations were observed for SNS. At a broader level, however, similar patterns were observed across activities, with associations tending to be concentrated among higher exposure groups. This suggests that prolonged engagement—particularly in activities such as online shopping and social networking—may characterize adolescents who spend extended time in relatively unstructured settings. Rather than reflecting direct content‐specific effects, these activities may be proxies for broader contextual and behavioral factors related to how adolescents spend unsupervised or individualized time online. Research on internet media use indicates that highly stable usage trajectories are more consistently linked to psychological difficulties^28^ and that adolescent risk behaviors arise from interacting individual, family, and community factors.^4^

This interpretation is consistent with prior research showing that adolescent substance use is associated with insufficient parental monitoring and weaker family structures,^4^ whereas improved family relationships and supervision are protective.^29^, ^30^ Unstructured leisure time and boredom have also been linked to alcohol and drug use,^31^ and unsupervised socializing increases risk behaviors beyond peer characteristics.^32^ In contrast, structured extracurricular activities may play a protective role.^33^ From this perspective, time spent on online shopping or auctions may be a proxy for shared contextual features such as limited supervision, individualized online engagement, and unstructured environments, rather than reflecting direct effects of shopping‐related content itself.

In this context, the consistent associations observed for online shopping or auctions should be interpreted cautiously. Importantly, the survey assessed time spent on an activity rather than purchasing behavior or financial expenditure. Among high school students, reported use likely reflects browsing, comparing items, or monitoring listings rather than direct consumption. Thus, the observed associations may not indicate exposure to specific shopping‐related content itself, but rather may function as proxies for broader contextual and behavioral factors, such as individualized online engagement, unstructured leisure time, and limited adult supervision.

However, given the cross‐sectional design, causality cannot be inferred. The association between media use and psychological problems may be bidirectional,^28^ and residual confounding due to unmeasured contextual factors cannot be excluded. Longitudinal and mixed‐method studies are required to clarify these mechanisms.

The reasons prolonged engagement in online shopping or auctions and SNS showed stronger and more consistent associations than other internet activities remain unclear and should be interpreted cautiously. Several explanations may be considered. First, different internet activities may involve varying degrees of parental monitoring or financial constraints. For example, activities such as online gaming or video streaming may require subscriptions or payments that are more likely to be supervised by parents, whereas browsing online shopping platforms may occur more independently. Second, the measurement of internet activities may have captured different underlying behaviors. For instance, “information searching” may include academic or school‐related activities, whereas searching for desired items may have been more likely to be reported under online shopping. This potential overlap in how activities were interpreted and reported by respondents may have contributed to the observed differences. Taken together, these findings may partly reflect differences in activity context and measurement rather than true differences in underlying mechanisms. Further research is needed to clarify these issues.

In low‐prevalence settings such as Japan, understanding how different types of online activities cluster with substance use may inform more nuanced surveillance and prevention strategies. This study adds to the literature by identifying engaging in online shopping or auctions and with SNS as potentially relevant activities. To date, research and public discourse have largely focused on the total duration of internet use or as a single aggregated exposure such as social media use, online gaming disorders, and online gambling.^23^, ^34^, ^35^, ^36^ Given our findings, which suggest that the associations between internet activities and substance‐use risk behaviors vary by activity type and tend to cluster among individuals with higher levels of engagement, assessing internet use not only in terms of total duration or as a single activity but also by distinguishing between different types of activities and evaluating them simultaneously, as well as by examining the time spent and content of each activity in greater detail, may provide additional insights.

### Limitations

This study had some limitations. First, the repeated measures cross‐sectional design precludes causal inference, and self‐reported measures may be subject to reporting bias. Estimates at extreme exposure levels were based on relatively small subgroups and may, therefore, be sensitive to sparse data bias. In addition, incomplete harmonization of the sex/gender items across survey waves required restriction of the primary analyses, which may limit generalizability, particularly for gender‐diverse adolescents.

A key limitation is the temporal mismatch between exposure and outcome measures. Substance use was assessed as any use within the past 12 months, whereas internet use reflected typical daily activities at or near the time of the survey. Accordingly, the observed associations should be interpreted strictly as cross‐sectional co‐occurrences rather than as evidence of temporal ordering. As internet use time was measured in six ordered categories, spline modeling was constrained by the discrete nature of exposure. Reverse causation (e.g., prior substance use influencing current internet use patterns) and contemporaneous clustering within shared behavioral contexts are both plausible.

Although exploratory sex‐stratified analyses showed broadly similar overall patterns across sexes, some activity‐specific differences were observed, particularly for illicit drug use. Therefore, the pooled estimates may partly mask heterogeneity in the associations between internet activities and substance use across sexes, and the findings should be interpreted cautiously.

Despite these limitations, this study provides descriptive evidence of how activity‐specific patterns of online engagement and substance‐use histories coexist at the population level in a low‐prevalence setting.

## CONCLUSION

Illicit drug use among Japanese high school students remained rare from 2018 to 2024, while alcohol and tobacco use declined. Associations between internet and substance use differed by activity type, indicating that patterns of online activities may provide more nuanced information than overall use.

## AUTHOR CONTRIBUTIONS

Takuya Shimane and Satomi Mizuno designed preliminary experiments and established the participant database. Takuya Shimane and Satomi Mizuno recruited participants and collected data. Takuya Shimane secured funding. Satomi Mizuno and Satoshi Inoura designed the study and performed statistical analyses. Satomi Mizuno drafted the manuscript. Takuya Shimane, Satoshi Inoura, Toshihiko Matsumoto, Maki Kitamura, Kunihiko Kitagaki, Akihiro Koide, and Kenji Takehara supervised the manuscript preparation. All authors revised and approved the final version of the manuscript for publication.

## CONFLICT OF INTEREST STATEMENT

The authors declare no conflicts of interest.

## ETHICS APPROVAL STATEMENT

This study was approved by the ethics committee of the National Center of Neurology and Psychiatry of Japan (approval numbers: A2018‐055 and A2023‐123). This study was conducted in accordance with the principles of the Declaration of Helsinki and STROBE reporting guidelines.

## PATIENT CONSENT STATEMENT

N/A.

## CLINICAL TRIAL REGISTRATION

N/A.

## Supporting information

Supporting File 1.

## Data Availability

The data that support the findings of this study are available from the corresponding author upon reasonable request and are subject to the completion of the appropriate institutional procedures.
