# Supplementary material for: Trends in substance use and associations with internet use among Japanese high school students: A nationwide survey, 2018–2024
Source: PCN Rep. 2026 Jun 17;5(2):e70365. doi: 10.1002/pcn5.70365 (PMC13273530; doi:10.1002/pcn5.70365)
Supplement: Supplementary file 1 — Supporting File 1. [file PCN5-5-e70365-s001.docx]

**Supporting information**

**Supplementary Table 1. Item-level counts (n) and unweighted percentages by survey year (2018, 2021, and 2024)**

|  | Total | 2018 | 2021 | 2024 | Missing |
| --- | --- | --- | --- | --- | --- |
|  | n = 142,567 (%) | n = 47,682 (%) | n = 43,998 (%) | n = 50,887 (%) | % |
| Grade |  |  |  |  | 0.0 |
| 10th | 57526 (40.4) | 17699 (37.1) | 17155 (39.0) | 22672 (44.6) |  |
| 11th | 47489 (33.3) | 16198 (34.0) | 14217 (32.3) | 17074 (33.6) |  |
| 12th | 37552 (26.3) | 13785 (28.9) | 12626 (28.7) | 11141 (21.9) |  |
| Sex |  |  |  |  | 0.0 |
| Male | 71734 (50.3) | 24662 (51.7) | 20978 (47.7) | 26094 (51.3) |  |
| Female | 70833 (49.7) | 23020 (48.3) | 23020 (52.3) | 24793 (48.7) |  |
| Lifestyle and school-related factors |  |  |  |  |  |
| Long unsupervised time | 23966 (16.9) | 7749 (16.4) | 7385 (16.9) | 8832 (17.4) | 0.7 |
| Lack of friends | 12134 (8.6) | 4389 (9.3) | 3532 (8.1) | 4213 (8.3) | 0.7 |
| Do not consult parents | 49287 (34.7) | 18076 (38.0) | 15143 (34.5) | 16068 (31.6) | 0.3 |
| Dissatisfaction with school life | 15998 (11.2) | 5978 (12.6) | 4990 (11.4) | 5030 (9.9) | 0.2 |
|  |  |  |  |  |  |
| Past-year substance use |  |  |  |  |  |
| Illicit drugs＊ | 311 (0.2) | 134 (0.3) | 68 (0.2) | 109 (0.2) | 0.0 |
| Alcohol | 18129 (12.7) | 8765 (18.4) | 5439 (12.4) | 3925 (7.7) | 0.0 |
| Tobacco | 2233 (1.6) | 1101 (2.3) | 511 (1.2) | 621 (1.2) | 0.0 |
|  |  |  |  |  |  |
| Time spent on SNS |  |  |  |  | 0.0 |
| No use (0 min) | 12162 (8.5) | 4328 (9.1) | 3887 (8.8) | 3947 (7.8) |  |
| Less than 30 min | 28329 (19.9) | 8695 (18.2) | 10459 (23.8) | 9175 (18.0) |  |
| About 1 h | 39053 (27.4) | 11627 (24.4) | 12969 (29.5) | 14457 (28.4) |  |
| About 2–3 h | 40205 (28.2) | 13959 (29.3) | 11051 (25.1) | 15195 (29.9) |  |
| About 4–5 h | 13354 (9.4) | 5215 (10.9) | 3240 (7.4) | 4899 (9.6) |  |
| 6 h or more | 9464 (6.6) | 3858 (8.1) | 2392 (5.4) | 3214 (6.3) |  |
| Time spent on online gaming |  |  |  |  | 0.0 |
| No use (0 min) | 53413 (37.5) | 17244 (36.2) | 17773 (40.4) | 18396 (36.2) |  |
| Less than 30 min | 23991 (16.8) | 7584 (15.9) | 7352 (16.7) | 9055 (17.8) |  |
| About 1 h | 27984 (19.6) | 9618 (20.2) | 7935 (18.0) | 10431 (20.5) |  |
| About 2–3 h | 24566 (17.2) | 8642 (18.1) | 7001 (15.9) | 8923 (17.5) |  |
| About 4–5 h | 6883 (4.8) | 2475 (5.2) | 2156 (4.9) | 2252 (4.4) |  |
| 6 h or more | 5730 (4.0) | 2119 (4.4) | 1781 (4.0) | 1830 (3.6) |  |
| Time spent on Internet searching/information browsing |  |  |  |  | 0.0 |
| No use (0 min) | 24673 (17.3) | 7531 (15.8) | 7285 (16.6) | 9857 (19.4) |  |
| Less than 30 min | 67491 (47.3) | 21747 (45.6) | 22031 (50.1) | 23713 (46.6) |  |
| About 1 h | 32686 (22.9) | 11616 (24.4) | 9809 (22.3) | 11261 (22.1) |  |
| About 2–3 h | 12228 (8.6) | 4700 (9.9) | 3334 (7.6) | 4194 (8.2) |  |
| About 4–5 h | 2744 (1.9) | 1043 (2.2) | 755 (1.7) | 946 (1.9) |  |
| 6 h or more | 2745 (1.9) | 1045 (2.2) | 784 (1.8) | 916 (1.8) |  |
| Time spent watching videos or listening to music online |  |  |  |  | 0.0 |
| No use (0 min) | 4118 (2.9) | 1555 (3.3) | 1112 (2.5) | 1451 (2.9) |  |
| Less than 30 min | 19404 (13.6) | 8006 (16.8) | 5608 (12.7) | 5790 (11.4) |  |
| About 1 h | 42807 (30.0) | 15264 (32.0) | 12945 (29.4) | 14598 (28.7) |  |
| About 2–3 h | 47727 (33.5) | 14903 (31.3) | 14994 (34.1) | 17830 (35.0) |  |
| About 4–5 h | 15774 (11.1) | 4386 (9.2) | 5160 (11.7) | 6228 (12.2) |  |
| 6 h or more | 12737 (8.9) | 3568 (7.5) | 4179 (9.5) | 4990 (9.8) |  |
| Time spent on online shopping or auctions |  |  |  |  | 0.0 |
| No use (0 min) | 88835 (62.3) | 31995 (67.1) | 26525 (60.3) | 30315 (59.6) |  |
| Less than 30 min | 38302 (26.9) | 11188 (23.5) | 12406 (28.2) | 14708 (28.9) |  |
| About 1 h | 10909 (7.7) | 3154 (6.6) | 3611 (8.2) | 4144 (8.1) |  |
| About 2–3 h | 3120 (2.2) | 932 (2.0) | 996 (2.3) | 1192 (2.3) |  |
| About 4–5 h | 603 (0.4) | 149 (0.3) | 205 (0.5) | 249 (0.5) |  |
| 6 h or more | 798 (0.6) | 264 (0.6) | 255 (0.6) | 279 (0.5) |  |
|  |  |  |  |  |  |
| Permissive attitudes toward illicit drug use | 6198 (4.3) | 1889 (4.0) | 1956 (4.4) | 2353 (4.6) | 0.0 |
| Permissive attitudes toward alcohol use | 34243 (24.2) | 13995 (29.5) | 10517 (24.1) | 9731 (19.3) | 0.7 |
| Permissive attitudes toward tobacco use | 9185 (6.6) | 3398 (7.4) | 2737 (6.3) | 3050 (6.0) | 1.9 |
|  |  |  |  |  |  |
| Geographic areas⁑ |  |  |  |  | 0.0 |
| Hokkaido–Tohoku | 16661 (11.7) | 5071 (10.6) | 4933 (11.2) | 6657 (13.1) |  |
| Kanto | 45856 (32.2) | 16023 (33.6) | 11662 (26.5) | 18171 (35.7) |  |
| Hokuriku–Tokai | 22022 (15.4) | 8066 (16.9) | 8002 (18.2) | 5954 (11.7) |  |
| Kinki | 24638 (17.3) | 7675 (16.1) | 7243 (16.5) | 9720 (19.1) |  |
| Chugoku–Shikoku | 15339 (10.8) | 6524 (13.7) | 5498 (12.5) | 3317 (6.5) |  |
| Kyushu–Okinawa | 18051 (12.7) | 4323 (9.1) | 6660 (15.1) | 7068 (13.9) |  |
|  |  |  |  |  |  |
| Survey mode |  |  |  |  |  |
| Paper-based questionnaire | 109643 (76.9) | 47682 (100.0) | 43998 (100.0) | 17963 (35.3) | 0.0 |

Abbreviations: SNS, social networking services.

Illicit drug use was defined as the use of cannabis, solvents, methamphetamine, new psychoactive substances (NPS), cocaine, or MDMA, all of which are prohibited by Japanese law.

⁑ The six geographic areas were as follows: Hokkaido–Tohoku (Hokkaido, Aomori, Iwate, Miyagi, Akita, Yamagata, Fukushima), Kanto (Ibaraki, Tochigi, Gunma, Saitama, Chiba, Tokyo, Kanagawa, Yamanashi, Nagano), and Hokuriku–Tokai (Niigata, Toyama, Ishikawa, Fukui, Gifu, Shizuoka, Aichi, and Mie).

Kinki (Shiga, Kyoto, Osaka, Hyogo, Nara, Wakayama); Chugoku–Shikoku (Tottori, Shimane, Okayama, Hiroshima, Yamaguchi, Tokushima, Kagawa, Ehime, Kochi); Kyushu–Okinawa (Fukuoka, Saga, Nagasaki, Kumamoto, Oita, Miyazaki, Kagoshima, Okinawa).

Missing values (%) indicate the proportion of missing responses for each item in the total sample.

**Supplementary Table 2. Construction of survey weights: definitions and formulas used for analysis**

| Level | Symbol | Formula / Value | Description |
| --- | --- | --- | --- |
| Stratum | Stratum (area) | 1-6 | Geographic strata used for sampling.  Area 1: Hokkaido–Tohoku;  Area 2: Kanto;  Area 3: Hokuriku–Tokai;  Area 4: Kinki;  Area 5: Chugoku–Shikoku;  Area 6: Kyushu–Okinawa. |
| School | students | Observed value | Number of students enrolled in each sampled school, obtained from the national school directory used for sampling. |
| School | Freq | Observed value | Total number of enrolled students in each geographic area (population size used for sampling). |
| School | p_1_ | students / Freq | Probability of school selection within each area, calculated as students/Freq using probability proportional to size. |
| Student | response | Observed value | Number of students who responded in each sampled school. |
| Student | p_2_ | response/students | Student-level response probability, calculated as the proportion of respondents among enrolled students. |
| Final | weight | 1/(p_1_ × p_2 )_ | Final survey weight, representing the inverse of the combined probability of school selection and student response. Weights were constructed separately for each survey year. |

**Supplementary Table 3. Distribution of final analysis weights: summary statistics**

| Statistic | Value |
| --- | --- |
| N (non-missing) | 142,567 |
| Mean | 8,924 |
| Standard Deviation (SD) | 21,223 |
| Minimum | 183 |
| 25th percentile (Q1) | 583 |
| Median | 1,485 |
| 75th percentile (Q3) | 9,832 |
| Maximum | 476,523 |

**Supplementary Table 4. Design effects, effective sample sizes, and clustering metrics by outcome**

| Outcome variable | Unweighted N | Design effect  (total) | Effective sample size | Design effect  (weighting) | Design effect  (clustering) | Intraclass correlation | Mean cluster size |
| --- | --- | --- | --- | --- | --- | --- | --- |
| Past-year illicit drug use | 142,567 | 5.50 | 25,906 | 6.66 | 0.83 | <0.001 | 513 |
| Past-year alcohol use | 142,567 | 28.81 | 4,949 | 6.66 | 4.33 | 0.007 | 513 |
| Past-year tobacco use | 142,567 | 16.71 | 8,531 | 6.66 | 2.51 | 0.003 | 513 |
| Lifetime illicit drug use | 142,567 | 10.98 | 12,985 | 6.66 | 1.65 | 0.001 | 513 |
| Time spent on social networking services | 142,567 | 91.58 | 1,557 | 6.66 | 13.76 | 0.025 | 513 |
| Time spent on online gaming | 142,567 | 124.47 | 1,145 | 6.66 | 18.70 | 0.035 | 513 |
| Time spent on Internet searching/information browsing | 142,567 | 37.00 | 3,853 | 6.66 | 5.56 | 0.009 | 513 |
| Time spent watching videos or listening to music online | 142,567 | 98.11 | 1,453 | 6.66 | 14.74 | 0.027 | 513 |
| Time spent on online shopping or auctions | 142,567 | 76.02 | 1,875 | 6.66 | 11.42 | 0.020 | 513 |

**Supplementary Table 5. Design-based weighted crude estimates (%) with 95% confidence intervals for substance use and Internet activity time categories, 2018–2024**

|  | 2018 | 2021 | 2024 |
| --- | --- | --- | --- |
| Past-year substance use |  |  |  |
| Illicit drugs | 0.4 (0.3–0.5) | 0.2 (0.1–0.2) | 0.2 (0.2–0.3) |
| Alcohol | 18.8 (17.0–20.5) | 12.5 (11.0–13.9) | 8.3 (7.5–9.1) |
| Tobacco | 2.8 (2.1–3.5) | 1.4 (1.0–1.8) | 1.5 (1.2–1.8) |
| Lifetime illicit drug use | 0.6 (0.4–0.7) | 0.2 (0.1–0.3) | 0.4 (0.2–0.5) |
| Time spent on social networking services |  |  |  |
| No use (0 min) | 9.6 (8.5–10.6) | 8.5 (7.7–9.3) | 7.9 (7.2–8.7) |
| Less than 30 min | 18.4 (16.4–20.4) | 22.3 (20.3–24.2) | 17.0 (15.7–18.3) |
| About 1 h | 23.5 (22.3–24.8) | 29.0 (27.8–30.3) | 27.2 (26.1–28.3) |
| About 2–3 h | 28.0 (26.3–29.7) | 25.7 (24.2–27.3) | 30.2 (28.9–31.5) |
| About 4–5 h | 11.2 (9.9–12.5) | 8.1 (6.9–9.4) | 10.3 (9.4–11.2) |
| 6 h or more | 9.3 (7.8–10.8) | 6.4 (5.2–7.6) | 7.4 (6.5–8.3) |
| Time spent on online gaming |  |  |  |
| No use (0 min) | 34.3 (31.6–37.0) | 38.5 (35.2–41.8) | 32.9 (30.5–35.2) |
| Less than 30 min | 15.3 (14.3–16.3) | 15.7 (14.9–16.5) | 17.1 (16.2–18.0) |
| About 1 h | 20.2 (19.3–21.1) | 17.9 (16.9–18.9) | 20.4 (19.4–21.3) |
| About 2–3 h | 18.7 (17.0–20.4) | 17.4 (15.8–18.9) | 19.4 (18.0–20.8) |
| About 4–5 h | 6.0 (5.0–7.0) | 5.6 (4.7–6.5) | 5.5 (4.8–6.3) |
| 6 h or more | 5.4 (4.2–6.6) | 5.0 (3.9–6.1) | 4.7 (3.9–5.6) |
| Time spent on Internet searching / information browsing |  |  |  |
| No use (0 min) | 15.6 (14.6–16.5) | 16.4 (15.4–17.5) | 19.0 (17.9–20.2) |
| Less than 30 min | 43.7 (42.0–45.4) | 49.0 (47.5–50.5) | 45.4 (44.0–46.7) |
| About 1 h | 25.0 (24.0–25.9) | 22.3 (21.5–23.1) | 22.6 (21.7–23.5) |
| About 2–3 h | 10.6 (9.7–11.5) | 8.2 (7.6–8.8) | 9.0 (8.4–9.6) |
| About 4–5 h | 2.4 (2.1–2.8) | 2.1 (1.8–2.4) | 2.0 (1.7–2.3) |
| 6 h or more | 2.7 (2.2–3.1) | 2.0 (1.6–2.3) | 2.0 (1.7–2.4) |
| Time spent watching videos or listening to music online |  |  |  |
| No use (0 min) | 3.2 (2.9–3.6) | 2.5 (2.2–2.8) | 2.8 (2.5–3.1) |
| Less than 30 min | 15.9 (14.1–17.6) | 11.4 (9.6–13.2) | 9.7 (8.8–10.6) |
| About 1 h | 30.9 (29.5–32.3) | 27.7 (26.0–29.4) | 27.1 (25.6–28.6) |
| About 2–3 h | 31.6 (30.3–32.8) | 34.4 (33.2–35.7) | 35.4 (34.5–36.4) |
| About 4–5 h | 9.7 (8.7–10.8) | 12.8 (11.6–13.9) | 13.1 (12.2–14.1) |
| 6 h or more | 8.7 (7.3–10.0) | 11.2 (9.4–12.9) | 11.8 (10.4–13.2) |
| Time spent on online shopping or auctions |  |  |  |
| No use (0 min) | 66.2 (63.3–69.0) | 58.5 (55.3–61.7) | 56.9 (54.6–59.2) |
| Less than 30 min | 23.5 (22.0–25.0) | 28.9 (27.3–30.5) | 30.0 (28.8–31.3) |
| About 1 h | 7.1 (6.0–8.1) | 8.9 (7.7–10.0) | 8.9 (8.0–9.8) |
| About 2–3 h | 2.2 (1.8–2.6) | 2.4 (2.0–2.9) | 2.8 (2.3–3.2) |
| About 4–5 h | 0.3 (0.2–0.4) | 0.5 (0.4–0.7) | 0.6 (0.5–0.8) |
| 6 h or more | 0.7 (0.5–0.8) | 0.7 (0.5–0.9) | 0.7 (0.5–0.9) |

Notes: All estimates are weighted crude prevalence (%) with 95% confidence intervals, accounting for the complex survey design (school-level clustering, area stratification, and sampling/non-response weights).

**Supplementary Table 6. Adjusted odds ratios for past-year illicit drug use by Internet use time category (Reference: no use [0 min]; survey-weighted restricted cubic spline model)**

| Internet activity | Time category | aOR (95% CI) vs no use |
| --- | --- | --- |
| Time spent on social networking services | No use (0 min) | Ref |
|  | Less than 30 min | 0.82 (0.37–1.79) |
|  | About 1 h | 0.70 (0.28–1.75) |
|  | About 2–3 h | 0.90 (0.39–2.04) |
|  | About 4–5 h | 1.82 (0.66–5.08) |
|  | 6 h or more | 2.25 (0.99–5.13) |
|  |  |  |
| Time spent on online gaming | No use (0 min) | Ref |
|  | Less than 30 min | 1.11 (0.57–2.14) |
|  | About 1 h | 0.77 (0.37–1.63) |
|  | About 2–3 h | 0.46 (0.21–1.02) |
|  | About 4–5 h | 0.46 (0.15–1.43) |
|  | 6 h or more | 1.74 (0.83–3.64) |
|  |  |  |
| Time spent on Internet searching / information browsing | No use (0 min) | Ref |
|  | Less than 30 min | 0.54 (0.33–0.91) |
|  | About 1 h | 0.71 (0.39–1.30) |
|  | About 2–3 h | 1.25 (0.62–2.51) |
|  | About 4–5 h | 1.64 (0.75–3.59) |
|  | 6 h or more | 3.08 (1.39–6.84) |
|  |  |  |
| Time spent watching videos or listening to music online | No use (0 min) | Ref |
|  | Less than 30 min | 0.51 (0.25–1.04) |
|  | About 1 h | 0.28 (0.14–0.59) |
|  | About 2–3 h | 0.26 (0.12–0.54) |
|  | About 4–5 h | 0.43 (0.15–1.24) |
|  | 6 h or more | 0.57 (0.28–1.14) |
|  |  |  |
| Time spent on online shopping or auctions | No use (0 min) | Ref |
|  | Less than 30 min | 1.45 (0.84–2.50) |
|  | About 1 h | 3.83 (1.82–8.05) |
|  | About 2–3 h | 5.58 (2.86–10.87) |
|  | About 4–5 h | 3.12 (0.63–15.54) |
|  | 6 h or more | 14.96 (6.38–35.09) |

**Outcome:** past-year illicit drug use.

**Supplementary Table 7. Adjusted odds ratios for past-year alcohol use by Internet use time category (Reference: no use [0 min]; survey-weighted restricted cubic spline model)**

| Internet activity | Time category | aOR (95% CI) vs no use |
| --- | --- | --- |
| Time spent on social networking services | No use (0 min) | Ref |
|  | Less than 30 min | 1.23 (1.04–1.46) |
|  | About 1 h | 1.60 (1.34–1.91) |
|  | About 2–3 h | 2.21 (1.84–2.65) |
|  | About 4–5 h | 3.02 (2.40–3.81) |
|  | 6 h or more | 3.62 (2.96–4.41) |
|  |  |  |
| Time spent on online gaming | No use (0 min) | Ref |
|  | Less than 30 min | 1.18 (1.04–1.34) |
|  | About 1 h | 1.32 (1.13–1.53) |
|  | About 2–3 h | 1.27 (1.06–1.54) |
|  | About 4–5 h | 1.17 (0.92–1.49) |
|  | 6 h or more | 1.77 (1.36–2.31) |
|  |  |  |
| Time spent on Internet searching / information browsing | No use (0 min) | Ref |
|  | Less than 30 min | 1.20 (1.03–1.39) |
|  | About 1 h | 1.29 (1.10–1.51) |
|  | About 2–3 h | 1.35 (1.16–1.57) |
|  | About 4–5 h | 1.58 (1.24–2.01) |
|  | 6 h or more | 2.05 (1.50–2.81) |
|  |  |  |
| Time spent watching videos or listening to music online | No use (0 min) | Ref |
|  | Less than 30 min | 0.89 (0.74–1.09) |
|  | About 1 h | 1.00 (0.80–1.25) |
|  | About 2–3 h | 1.21 (0.97–1.51) |
|  | About 4–5 h | 1.41 (1.10–1.81) |
|  | 6 h or more | 1.97 (1.51–2.56) |
|  |  |  |
| Time spent on online shopping or auctions | No use (0 min) | Ref |
|  | Less than 30 min | 1.82 (1.65–2.01) |
|  | About 1 h | 2.64 (2.30–3.03) |
|  | About 2–3 h | 2.73 (2.20–3.38) |
|  | About 4–5 h | 2.48 (1.58–3.89) |
|  | 6 h or more | 4.84 (3.13–7.47) |

**Outcome:** past-year alcohol use.

**Supplementary Table 8. Adjusted odds ratios for past-year tobacco use by Internet use time category (Reference: no use [0 min]; survey-weighted restricted cubic spline model)**

| Internet activity | Time category | aOR (95% CI) vs no use |
| --- | --- | --- |
| Time spent on social networking services | No use (0 min) | Ref |
|  | Less than 30 min | 0.71 (0.47–1.06) |
|  | About 1 h | 1.08 (0.70–1.67) |
|  | About 2–3 h | 1.98 (1.30–3.02) |
|  | About 4–5 h | 2.47 (1.53–3.97) |
|  | 6 h or more | 4.08 (2.85–5.85) |
|  |  |  |
| Time spent on online gaming | No use (0 min) | Ref |
|  | Less than 30 min | 1.12 (0.84–1.48) |
|  | About 1 h | 1.13 (0.81–1.58) |
|  | About 2–3 h | 0.91 (0.63–1.31) |
|  | About 4–5 h | 0.71 (0.42–1.18) |
|  | 6 h or more | 1.41 (0.90–2.23) |
|  |  |  |
| Time spent on Internet searching / information browsing | No use (0 min) | Ref |
|  | Less than 30 min | 0.95 (0.73–1.24) |
|  | About 1 h | 0.85 (0.64–1.13) |
|  | About 2–3 h | 0.96 (0.77–1.19) |
|  | About 4–5 h | 1.55 (1.01–2.38) |
|  | 6 h or more | 2.01 (1.29–3.12) |
|  |  |  |
| Time spent watching videos or listening to music online | No use (0 min) | Ref |
|  | Less than 30 min | 0.53 (0.34–0.81) |
|  | About 1 h | 0.47 (0.30–0.75) |
|  | About 2–3 h | 0.62 (0.41–0.93) |
|  | About 4–5 h | 0.85 (0.55–1.30) |
|  | 6 h or more | 0.96 (0.62–1.50) |
|  |  |  |
| Time spent on online shopping or auctions | No use (0 min) | Ref |
|  | Less than 30 min | 2.30 (1.75–3.04) |
|  | About 1 h | 4.37 (3.25–5.89) |
|  | About 2–3 h | 5.83 (4.21–8.07) |
|  | About 4–5 h | 5.90 (2.98–11.65) |
|  | 6 h or more | 8.91 (4.75–16.74) |

**Outcome:** past-year tobacco use.

**Supplementary Table 9. Category-specific adjusted odds ratios for past-year illicit drug use by time spent on Internet activities (reference: no use)**

| Internet activity | Time category  (ref: No use) | Unweighted n | aOR (95% CI) vs no use | p-value |
| --- | --- | --- | --- | --- |
| Time spent on social networking services | No use (0 min) | 12,162 | Ref |  |
|  | Less than 30 min | 28,329 | 0.54 (0.23–1.23) | 0.142 |
|  | About 1 h | 39,053 | 0.83 (0.36–1.89) | 0.655 |
|  | About 2–3 h | 40,205 | 0.64 (0.26–1.53) | 0.314 |
|  | About 4–5 h | 13,354 | 1.85 (0.74–4.62) | 0.191 |
|  | 6 h or more | 9,464 | 1.96 (0.89–4.34) | 0.097 |
|  |  |  |  |  |
| Time spent on online gaming | No use (0 min) | 53,413 | Ref |  |
|  | Less than 30 min | 23,991 | 1.17 (0.57–2.41) | 0.672 |
|  | About 1 h | 27,984 | 0.73 (0.33–1.63) | 0.442 |
|  | About 2–3 h | 24,566 | 0.50 (0.20–1.22) | 0.130 |
|  | About 4–5 h | 6,883 | 0.42 (0.13–1.40) | 0.159 |
|  | 6 h or more | 5,730 | 1.77 (0.83–3.79) | 0.144 |
|  |  |  |  |  |
| Time spent on Internet searching / information browsing | No use (0 min) | 26,673 | Ref |  |
|  | Less than 30 min | 67,491 | 0.63 (0.36–1.10) | 0.105 |
|  | About 1 h | 32,686 | 0.57 (0.29–1.13) | 0.108 |
|  | About 2–3 h | 12,228 | 1.61 (0.74–3.52) | 0.233 |
|  | About 4–5 h | 2,744 | 1.13 (0.37–3.46) | 0.826 |
|  | 6 h or more | 2,745 | 3.28 (1.50–7.18) | 0.003 |
|  |  |  |  |  |
| Time spent watching videos or listening to music online | No use (0 min) | 4,118 | Ref |  |
|  | Less than 30 min | 19,404 | 0.46 (0.19–1.10) | 0.083 |
|  | About 1 h | 42,807 | 0.29 (0.14–0.60) | <0.001 |
|  | About 2–3 h | 47,727 | 0.24 (0.10–0.56) | <0.001 |
|  | About 4–5 h | 15,774 | 0.43 (0.15–1.20) | 0.109 |
|  | 6 h or more | 12,737 | 0.55 (0.27–1.12) | 0.101 |
|  |  |  |  |  |
| Time spent on online shopping or auctions | No use (0 min) | 88,835 | Ref |  |
|  | Less than 30 min | 38,302 | 1.44 (0.80–2.59) | 0.226 |
|  | About 1 h | 10,909 | 3.87 (1.75–8.57) | <0.001 |
|  | About 2–3 h | 3,120 | 5.46 (2.37–12.58) | <0.001 |
|  | About 4–5 h | 603 | 3.32 (0.69–15.92) | 0.135 |
|  | 6 h or more | 798 | 14.92 (6.30–35.32) | <0.001 |

Outcome: past-year illicit drug use.

Models were estimated using survey-weighted logistic regression (svyglm, quasibinomial).

The models were adjusted for survey year (categorical; reference: 2024), grade, sex, area, and survey mode (paper/online).

The survey design accounted for clustering by school (PSU), stratification by area, and sampling weights (survey.lonely.psu = "adjust").

Category-specific estimates may be unstable because of the low prevalence of outcomes, particularly in higher-exposure categories.

Unweighted n indicates the number of participants with non-missing values for both the outcome and corresponding Internet activity variable within the analysis sample.

The overall dose–response patterns were assessed using pre-specified spline models (Figure 2; Supplementary Tables 6–8) and summarized using ordinal trend models (Table 4).

**Supplementary Table 10. Category-specific adjusted odds ratios for past-year alcohol use by time spent on Internet activities (reference: no use)**

| Internet activity | Time category  (ref: No use) | Unweighted n | aOR (95% CI) vs no use | p-value |
| --- | --- | --- | --- | --- |
| Time spent on social networking services | No use (0 min) | 12,162 | Ref |  |
|  | Less than 30 min | 28,329 | 1.11 (0.90–1.38) | 0.326 |
|  | About 1 h | 39,053 | 1.61 (1.36–1.91) | <0.001 |
|  | About 2–3 h | 40,205 | 2.07 (1.69–2.53) | <0.001 |
|  | About 4–5 h | 13,354 | 2.97 (2.37–3.72) | <0.001 |
|  | 6 h or more | 9,464 | 3.46 (2.82–4.25) | <0.001 |
|  |  |  |  |  |
| Time spent on online gaming | No use (0 min) | 53,413 | Ref |  |
|  | Less than 30 min | 23,991 | 1.15 (1.00–1.32) | 0.054 |
|  | About 1 h | 27,984 | 1.34 (1.13–1.60) | 0.001 |
|  | About 2–3 h | 24,566 | 1.24 (1.03–1.51) | 0.027 |
|  | About 4–5 h | 6,883 | 1.20 (0.94–1.54) | 0.139 |
|  | 6 h or more | 5,730 | 1.76 (1.35–2.28) | <0.001 |
|  |  |  |  |  |
| Time spent on Internet searching / information browsing | No use (0 min) | 24,673 | Ref |  |
|  | Less than 30 min | 67,491 | 1.21 (1.04–1.40) | 0.014 |
|  | About 1 h | 32,686 | 1.28 (1.07–1.52) | 0.007 |
|  | About 2–3 h | 12,228 | 1.37 (1.17–1.60) | <0.001 |
|  | About 4–5 h | 27,44 | 1.55 (1.19–2.02) | 0.001 |
|  | 6 h or more | 2,745 | 2.06 (1.49–2.85) | <0.001 |
|  |  |  |  |  |
| Time spent watching videos or listening to music online | No use (0 min) | 4,118 | Ref |  |
|  | Less than 30 min | 19,404 | 0.97 (0.75–1.25) | 0.810 |
|  | About 1 h | 42,807 | 1.02 (0.80–1.29) | 0.886 |
|  | About 2–3 h | 47,727 | 1.27 (1.00–1.62) | 0.051 |
|  | About 4–5 h | 15,774 | 1.45 (1.12–1.88) | 0.005 |
|  | 6 h or more | 12,737 | 2.05 (1.54–2.72) | <0.001 |
|  |  |  |  |  |
| Time spent on online shopping or auctions | No use (0 min) | 88,835 | Ref |  |
|  | Less than 30 min | 38,302 | 1.82 (1.65–2.00) | <0.001 |
|  | About 1 h | 10,909 | 2.64 (2.28–3.06) | <0.001 |
|  | About 2–3 h | 3,120 | 2.73 (2.07–3.59) | <0.001 |
|  | About 4–5 h | 603 | 2.48 (1.48–4.16) | <0.001 |
|  | 6 h or more | 798 | 4.84 (3.11–7.52) | <0.001 |

Outcome: past-year alcohol use.

Models were estimated using survey-weighted logistic regression (svyglm, quasibinomial).

The models were adjusted for survey year (categorical; reference: 2024), grade, sex, area, and survey mode (paper/online).

The survey design accounted for clustering by school (PSU), stratification by area, and sampling weights (survey.lonely.psu = "adjust").

Category-specific estimates may be unstable in extreme-exposure categories because of the small numbers in some time-use categories.

Unweighted n indicates the number of participants with non-missing values for both the outcome and corresponding Internet activity variable within the analysis sample.

The overall dose–response patterns were assessed using pre-specified spline models (Figure 2; Supplementary Tables 6–8) and summarized using ordinal trend models (Table 4).

**Supplementary Table 11. Category-specific adjusted odds ratios for past-year tobacco smoking by time spent on Internet activities (reference: no use)**

| Internet activity | Time category  (ref: No use) | Unweighted n | aOR (95% CI) vs no use | p-value |
| --- | --- | --- | --- | --- |
| Time spent on social networking services | No use (0 min) | 12,162 | Ref |  |
|  | Less than 30 min | 28,329 | 0.69 (0.42–1.14) | 0.147 |
|  | About 1 h | 39,053 | 1.09 (0.70–1.70) | 0.706 |
|  | About 2–3 h | 40,205 | 1.95 (1.27–2.99) | 0.003 |
|  | About 4–5 h | 13,354 | 2.46 (1.54–3.94) | <0.001 |
|  | 6 h or more | 9,464 | 4.04 (2.85–5.74) | <0.001 |
|  |  |  |  |  |
| Time spent on online gaming | No use (0 min) | 53,413 | Ref |  |
|  | Less than 30 min | 23,991 | 1.14 (0.87–1.49) | 0.336 |
|  | About 1 h | 27,984 | 1.11 (0.74–1.67) | 0.609 |
|  | About 2–3 h | 24,566 | 0.93 (0.66–1.31) | 0.677 |
|  | About 4–5 h | 6,883 | 0.69 (0.39–1.21) | 0.197 |
|  | 6 h or more | 5,730 | 1.42 (0.91–2.22) | 0.122 |
|  |  |  |  |  |
| Time spent on Internet searching / information browsing | No use (0 min) | 24,673 | Ref |  |
|  | Less than 30 min | 67,491 | 0.94 (0.71–1.25) | 0.670 |
|  | About 1 h | 32,686 | 0.87 (0.66–1.14) | 0.324 |
|  | About 2–3 h | 12,228 | 0.91 (0.65–1.27) | 0.570 |
|  | About 4–5 h | 2,744 | 1.62 (1.03–2.55) | 0.036 |
|  | 6 h or more | 2,745 | 1.99 (1.26–3.12) | 0.003 |
|  |  |  |  |  |
| Time spent watching videos or listening to music online | No use (0 min) | 4,118 | Ref |  |
|  | Less than 30 min | 19,404 | 0.49 (0.28–0.86) | 0.013 |
|  | About 1 h | 42,807 | 0.47 (0.30–0.75) | 0.001 |
|  | About 2–3 h | 47,727 | 0.60 (0.40–0.89) | 0.013 |
|  | About 4–5 h | 15,774 | 0.84 (0.55–1.27) | 0.403 |
|  | 6 h or more | 12,737 | 0.94 (0.60–1.48) | 0.799 |
|  |  |  |  |  |
| Time spent on online shopping or auctions | No use (0 min) | 88,835 | Ref |  |
|  | Less than 30 min | 38,302 | 2.29 (1.74–3.01) | <0.001 |
|  | About 1 h | 10,909 | 4.44 (3.21–6.13) | <0.001 |
|  | About 2–3 h | 3,120 | 5.66 (3.77–8.47) | <0.001 |
|  | About 4–5 h | 603 | 6.20 (2.90–13.25) | <0.001 |
|  | 6 h or more | 798 | 8.86 (4.73–16.60) | <0.001 |

Outcome: past-year tobacco use.

Models were estimated using survey-weighted logistic regression (svyglm, quasibinomial).

The models were adjusted for survey year (categorical; reference: 2024), grade, sex, area, and survey mode (paper/online).

The survey design accounted for clustering by school (PSU), stratification by area, and sampling weights (survey.lonely.psu = "adjust").

Category-specific estimates may be unstable, particularly in higher exposure categories, owing to small numbers in some categories.

Unweighted n indicates the number of participants with non-missing values for both the outcome and corresponding Internet activity variable within the analysis sample.

The overall dose–response patterns were assessed using pre-specified spline models (Figure 2; Supplementary Tables 6–8) and summarized using ordinal trend models (Table 4).

**Supplementary Table 12. Comparison of linear trend and restricted cubic spline (RCS) models for associations between Internet activities and substance use**

| Outcome | Internet activity | Linear trend p-value | RCS overall association p-value | Non-linearity p-value |
| --- | --- | --- | --- | --- |
| Illicit drugs | SNS | 0.014 | 0.008 | 0.202 |
|  | Online gaming | 0.655 | 0.023 | 0.012 |
|  | Internet searching | 0.005 | <0.001 | 0.012 |
|  | Video/music | 0.881 | 0.004 | 0.002 |
|  | Online shopping | <0.001 | <0.001 | 0.505 |
|  |  |  |  |  |
| alcohol | SNS | <0.001 | <0.001 | 0.434 |
|  | Online gaming | <0.001 | <0.001 | 0.022 |
|  | Internet searching | <0.001 | <0.001 | 0.379 |
|  | Video/music | <0.001 | <0.001 | 0.012 |
|  | Online shopping | <0.001 | <0.001 | <0.001 |
|  |  |  |  |  |
| tobacco | SNS | <0.001 | <0.001 | 0.011 |
|  | Online gaming | 0.995 | 0.045 | 0.025 |
|  | Internet searching | 0.044 | 0.004 | 0.008 |
|  | Video/music | 0.002 | <0.001 | <0.001 |
|  | Online shopping | <0.001 | <0.001 | <0.001 |

Nonlinear p-values were obtained from design-based Wald tests comparing the full RCS model with a reduced linear model.

Linear trend p-values were derived from models treating Internet use as a single ordinal predictor (0–5).

RCS models use restricted cubic splines with linear tails implemented via natural cubic spline basis functions.

All models accounted for the complex survey design and were adjusted for the survey year, grade, sex, geographic area, and survey mode. SNS, social networking services.

**Supplementary Table 13. Benjamini–Hochberg false discovery rate adjustment for prespecified primary spline-based overall association tests**

| Outcome | Internet activity | RCS overall p | BH-FDR q |
| --- | --- | --- | --- |
| Illicit drugs | Time spent on SNS | 0.008 | 0.009 |
|  | Time spent on online gaming | 0.023 | 0.025 |
|  | Time spent on Internet searching / information browsing | <0.001 | <0.001 |
|  | Time spent watching videos or listening to music online | 0.004 | 0.005 |
|  | Time spent on online shopping or auctions | <0.001 | <0.001 |
|  |  |  |  |
| Alcohol | Time spent on SNS | <0.001 | <0.001 |
|  | Time spent on online gaming | <0.001 | <0.001 |
|  | Time spent on Internet searching / information browsing | <0.001 | <0.001 |
|  | Time spent watching videos or listening to music online | <0.001 | <0.001 |
|  | Time spent on online shopping or auctions | <0.001 | <0.001 |
|  |  |  |  |
| Tobacco | Time spent on SNS | <0.001 | <0.001 |
|  | Time spent on online gaming | 0.045 | 0.045 |
|  | Time spent on Internet searching / information browsing | 0.004 | 0.005 |
|  | Time spent watching videos or listening to music online | <0.001 | <0.001 |
|  | Time spent on online shopping or auctions | <0.001 | <0.001 |

The RCS overall p-values were obtained from design-based Wald tests of the spline terms in the prespecified survey-weighted restricted cubic spline models adjusted for survey year, grade, sex, geographic area, and survey mode, accounting for sampling weights, stratification, and clustering at the school level.

BH-FDR q-values were computed across 15 pre-specified primary tests (five Internet activities × three outcomes) using the Benjamini–Hochberg procedure. Values are displayed as “<0.001” when p or q < 0.001. SNS, social networking services.

**Supplementary Table 14. Sensitivity analysis: associations between Internet use patterns and past-year illicit drug, alcohol, and tobacco use among Japanese high school students (trend model)**

|  | Illicit drugs | | Alcohol | | Tobacco | |
| --- | --- | --- | --- | --- | --- | --- |
| Internet activity | aOR per 1-category increase (95% CI) | p-value | aOR per 1-category increase (95% CI) | p-value | aOR per 1-category increase (95% CI) | p-value |
| SNS | 1.15 (0.97–1.37) | 0.098 | 1.18 (1.14–1.22) | <0.001 | 1.25 (1.13–1.38) | <0.001 |
| Online gaming | 0.94 (0.81–1.11) | 0.478 | 1.03 (0.98–1.08) | 0.239 | 0.94 (0.85–1.03) | 0.174 |
| Internet searching / information browsing | 1.13 (0.95–1.33) | 0.163 | 1.07 (1.02–1.13) | 0.004 | 0.99 (0.93–1.06) | 0.822 |
| Video/music | 0.92 (0.77–1.09) | 0.335 | 1.09 (1.04–1.13) | <0.001 | 1.01 (0.92–1.11) | 0.794 |
| Online shopping or auctions | 1.46 (1.25–1.71) | <0.001 | 1.28 (1.22–1.34) | <0.001 | 1.40 (1.29–1.52) | <0.001 |

**Abbreviations**: aOR, adjusted odds ratio; CI, confidence interval; SNS, social networking services.

**Outcome variables**: Past-year illicit drug use, alcohol use, and tobacco use (yes/no).

**Model:** Survey-weighted logistic regression (SVYGLM, quasibinomial). Internet use variables were entered as 6-level ordinal predictors (0–5), and aORs represented the change in odds per one-category increase.

**Adjustment (sensitivity):** Survey year (categorical; reference = 2024), grade, sex, area, survey mode, permissive attitudes toward substance use, school dissatisfaction, lack of close friends, limited parental consultation, and long unsupervised time.

**Survey design:** clustering by school (PSU), stratification by area, sampling weights applied; survey.lonely.psu = "adjust.”

This table replicates the ordinal trend models shown in Table 4 with additional adjustments for psychosocial and behavioral covariates to assess the robustness of the primary findings.

**Supplementary Table 15. Sensitivity analysis: category-specific adjusted odds ratios for past-year illicit drug use by time spent on Internet activities (reference: no use)**

| Internet activity | Time category  (ref: No use) | aOR (95% CI) vs no use |
| --- | --- | --- |
| Time spent on social networking services | No use (0 min) | Ref |
|  | Less than 30 min | 0.47 (0.21–1.08) |
|  | About 1 h | 0.74 (0.34–1.63) |
|  | About 2–3 h | 0.56 (0.24–1.31) |
|  | About 4–5 h | 1.46 (0.60–3.60) |
|  | 6 h or more | 1.24 (0.55–2.82) |
|  |  |  |
| Time spent on online gaming | No use (0 min) | Ref |
|  | Less than 30 min | 1.19 (0.59–2.43) |
|  | About 1 h | 0.78 (0.36–1.73) |
|  | About 2–3 h | 0.54 (0.23–1.28) |
|  | About 4–5 h | 0.44 (0.13–1.51) |
|  | 6 h or more | 1.35 (0.63–2.90) |
|  |  |  |
| Time spent on Internet searching / information browsing | No use (0 min) | Ref |
|  | Less than 30 min | 0.63 (0.35–1.14) |
|  | About 1 h | 0.55 (0.27–1.09) |
|  | About 2–3 h | 1.18 (0.53–2.60) |
|  | About 4–5 h | 0.77 (0.24–2.45) |
|  | 6 h or more | 1.76 (0.78–3.95) |
|  |  |  |
| Time spent watching videos or listening to music online | No use (0 min) | Ref |
|  | Less than 30 min | 0.63 (0.25–1.61) |
|  | About 1 h | 0.39 (0.18–0.83) |
|  | About 2–3 h | 0.30 (0.12–0.76) |
|  | About 4–5 h | 0.44 (0.15–1.25) |
|  | 6 h or more | 0.52 (0.25–1.10) |
|  |  |  |
| Time spent on online shopping or auctions | No use (0 min) | Ref |
|  | Less than 30 min | 1.29 (0.70–2.36) |
|  | About 1 h | 2.99 (1.40–6.36) |
|  | About 2–3 h | 4.44 (1.80–10.96) |
|  | About 4–5 h | 2.10 (0.46–9.59) |
|  | 6 h or more | 5.47 (1.97–15.21) |

**Model**: Survey-weighted logistic regression (svyglm, quasi-binomial) with Internet use entered as a categorical variable (reference: no use [0 min]).
**Adjustment**: Same as Supplementary Table 9 (additional adjustment for perception of drug use and lifestyle/school-related factors plus the main covariates).
**Note**: Category-specific estimates may be unstable, particularly in higher-exposure categories, owing to small numbers in some categories.

**Supplementary Table 16. Associations between Internet use patterns and past-year alcohol drinking among Japanese high school students**

| Internet activity | Time category  (ref: No use) | aOR (95% CI) vs no use |
| --- | --- | --- |
| Time spent on social networking services | No use (0 min) | Ref |
|  | Less than 30 min | 0.99 (0.79–1.24) |
|  | About 1 h | 1.35 (1.13–1.62) |
|  | About 2–3 h | 1.59 (1.28–1.98) |
|  | About 4–5 h | 1.92 (1.54–2.38) |
|  | 6 h or more | 1.98 (1.61–2.44) |
|  |  |  |
| Time spent on online gaming | No use (0 min) | Ref |
|  | Less than 30 min | 1.11 (0.96–1.27) |
|  | About 1 h | 1.37 (1.12–1.66) |
|  | About 2–3 h | 1.14 (0.94–1.37) |
|  | About 4–5 h | 0.90 (0.71–1.15) |
|  | 6 h or more | 1.29 (0.98–1.69) |
|  |  |  |
| Time spent on Internet searching / information browsing | No use (0 min) | Ref |
|  | Less than 30 min | 1.26 (1.06–1.49) |
|  | About 1 h | 1.29 (1.06–1.58) |
|  | About 2–3 h | 1.24 (1.01–1.53) |
|  | About 4–5 h | 1.49 (1.06–2.09) |
|  | 6 h or more | 1.57 (1.09–2.27) |
|  |  |  |
| Time spent watching videos or listening to music online | No use (0 min) | Ref |
|  | Less than 30 min | 1.01 (0.74–1.39) |
|  | About 1 h | 1.00 (0.73–1.37) |
|  | About 2–3 h | 1.12 (0.81–1.53) |
|  | About 4–5 h | 1.10 (0.79–1.52) |
|  | 6 h or more | 1.44 (1.03–2.02) |
|  |  |  |
| Time spent on online shopping or auctions | No use (0 min) | Ref |
|  | Less than 30 min | 1.46 (1.33–1.62) |
|  | About 1 h | 1.87 (1.61–2.17) |
|  | About 2–3 h | 1.75 (1.31–2.35) |
|  | About 4–5 h | 1.79 (0.99–3.23) |
|  | 6 h or more | 3.53 (2.16–5.77) |

**Model**: Survey-weighted logistic regression (svyglm, quasi-binomial) with Internet use entered as a categorical variable (reference: no use [0 min]).
**Adjustment**: Same as in Supplementary Table 10 (with additional adjustment for permissive attitudes toward alcohol use and psychosocial/lifestyle covariates plus the main covariates).
**Note**: Category-specific estimates may be unstable, particularly in higher-exposure categories, owing to small numbers in some categories.

**Supplementary Table 17. Category-specific adjusted odds ratios for past-year tobacco smoking by time spent on Internet activities (reference: no use)**

| Internet activity | Time category  (ref: No use) | aOR (95% CI) vs no use |
| --- | --- | --- |
| Time spent on social networking services | No use (0 min) | Ref |
|  | Less than 30 min | 0.625 (0.367–1.066) |
|  | About 1 h | 0.957 (0.604–1.515) |
|  | About 2–3 h | 1.455 (0.918–2.307) |
|  | About 4–5 h | 1.359 (0.778–2.376) |
|  | 6 h or more | 2.117 (1.407–3.186) |
|  |  |  |
| Time spent on online gaming | No use (0 min) | Ref |
|  | Less than 30 min | 1.159 (0.847–1.585) |
|  | About 1 h | 1.207 (0.791–1.844) |
|  | About 2–3 h | 0.845 (0.585–1.220) |
|  | About 4–5 h | 0.520 (0.309–0.875) |
|  | 6 h or more | 0.931 (0.555–1.564) |
|  |  |  |
| Time spent on Internet searching / information browsing | No use (0 min) | Ref |
|  | Less than 30 min | 1.044 (0.784–1.391) |
|  | About 1 h | 0.892 (0.661–1.204) |
|  | About 2–3 h | 0.712 (0.466–1.089) |
|  | About 4–5 h | 1.356 (0.881–2.087) |
|  | 6 h or more | 1.318 (0.838–2.074) |
|  |  |  |
| Time spent watching videos or listening to music online | No use (0 min) | Ref |
|  | Less than 30 min | 0.583 (0.318–1.069) |
|  | About 1 h | 0.510 (0.310–0.839) |
|  | About 2–3 h | 0.574 (0.354–0.929) |
|  | About 4–5 h | 0.665 (0.404–1.095) |
|  | 6 h or more | 0.629 (0.367–1.080) |
|  |  |  |
| Time spent on online shopping or auctions | No use (0 min) | Ref |
|  | Less than 30 min | 1.562 (1.138–2.144) |
|  | About 1 h | 2.600 (1.864–3.626) |
|  | About 2–3 h | 2.798 (1.741–4.497) |
|  | About 4–5 h | 3.319 (1.617–6.815) |
|  | 6 h or more | 4.459 (2.534–7.844) |

**Model**: Survey-weighted logistic regression (svyglm, quasi-binomial) with Internet use entered as a categorical variable (reference: no use [0 min]).
**Adjustment**: Same as in Supplementary Table 11 (with additional adjustment for permissive attitudes toward tobacco use and psychosocial/lifestyle covariates plus the main covariates).
**Note**: Category-specific estimates may be unstable, particularly in higher-exposure categories, owing to small numbers in some categories.

**Supplementary Table 18. Sex-stratified trend models of associations between Internet use patterns and past-year substance use among Japanese high school students**

|  |  | Male | | Female | |
| --- | --- | --- | --- | --- | --- |
| Outcome | Internet activity | aOR per 1-category increase (95% CI) | p-value | aOR per 1-category increase (95% CI) | p-value |
| Illicit drugs | SNS | 1.24 (1.04–1.47) | 0.017 | 1.31 (0.84–2.04) | 0.232 |
|  | Online gaming | 0.89 (0.74–1.08) | 0.246 | 1.35 (1.05–1.73) | 0.019 |
|  | Internet searching / information browsing | 1.33 (1.10–1.61) | 0.004 | 1.10 (0.74–1.61) | 0.646 |
|  | Video/music | 0.97 (0.80–1.18) | 0.771 | 1.07 (0.68–1.68) | 0.769 |
|  | Online shopping or auctions | 1.72 (1.48–1.99) | <0.001 | 1.82 (1.29–2.56) | <0.001 |
|  |  |  |  |  |  |
| Alcohol | SNS | 1.29 (1.24–1.35) | <0.001 | 1.35 (1.28–1.43) | <0.001 |
|  | Online gaming | 1.09 (1.03–1.15) | 0.001 | 1.08 (1.01–1.16) | 0.027 |
|  | Internet searching / information browsing | 1.12 (1.05–1.19) | <0.001 | 1.12 (1.05–1.19) | <0.001 |
|  | Video/music | 1.19 (1.12–1.25) | <0.001 | 1.22 (1.15–1.30) | <0.001 |
|  | Online shopping or auctions | 1.37 (1.29–1.47) | <0.001 | 1.50 (1.40–1.62) | <0.001 |
|  |  |  |  |  |  |
| Tobacco | SNS | 1.40 (1.29–1.53) | <0.001 | 1.64 (1.31–2.05) | <0.001 |
|  | Online gaming | 0.96 (0.87–1.06) | 0.426 | 1.13 (0.98–1.30) | 0.094 |
|  | Internet searching / information browsing | 1.06 (0.97–1.16) | 0.208 | 1.14 (1.01–1.29) | 0.029 |
|  | Video/music | 1.12 (1.02–1.22) | 0.012 | 1.26 (1.01–1.59) | 0.045 |
|  | Online shopping or auctions | 1.62 (1.50–1.76) | <0.001 | 1.75 (1.51–2.04) | <0.001 |

**Abbreviations**: aOR, adjusted odds ratio; 95% CI, 95% confidence interval; SNS, social networking services.
Outcome variables were: past-year illicit drug use, alcohol use, and tobacco use.

Model: survey-weighted logistic regression (svyglm, quasibinomial).

Exposure variables: Time spent on Internet activities was entered as an ordinal predictor (six ordered categories).

Interpretation: aORs represent the change in the odds of substance use per 1-category increase in time spent on the activity.

Covariates: Survey year (categorical; reference = 2024), grade, area, and survey mode (paper/online).

Survey design: clustered by school (PSU), stratified by area, with sampling weights applied (survey.lonely.psu = "adjust").

Sex-stratified analyses were conducted to explore potential gender differences in the associations between Internet activities and substance use.
